# Surface Reaction-Mediated Nanomolar Detection of Chemically Activated Plant Metabolite Biomarkers with Field Effect Sensors

**DOI:** 10.1021/acs.langmuir.6c05228

**Published:** 2026-10-29

**Authors:** Sehyun Park, Jinyoung Kim, Jinyoung Choi, Jaejun Lee, Vladimir V. Tsukruk

**Affiliations:** School of Materials Science and Engineering, 1372 Georgia Institute of Technology, Atlanta, Georgia 30332, United States

## Abstract

Among potent psychedelic drugs derived from plant metabolites, *N*,*N*-dimethyltryptamine (DMT) is a known endogenous tertiary amine alkaloid that can be harmful to brain activity even when present in biological fluids at nanomolar concentrations. Conventional electrochemical detection methods reach only micromolar limits, far above the physiological window, while full mass-spectrometric analysis needed for practical nanomolar detection depends on centralized medical facilities. Here, we present a three-step sensing strategy combining selective chemical activation with a self-assembled monolayer (SAM)-functionalized surface in order to facilitate nanomolar detection with extended-gate field-effect transistor (EGFET) sensors. Initially, the target tertiary amine is selectively oxidized to its *N*-oxide and rearranged to the corresponding amide, introducing a reactive handle absent from the native molecule. As a next step, the activated analyte is captured by an amino-terminated SAM on the gate electrode of the FET device. Finally, the resulting shift in gate surface potential is transduced by the EGFET into a detectable electrical output. Using gramine as a DMT surrogate, the fabricated sensory device achieves a limit of detection of 7 nM, well within the required physiological window. The surface-bound amount was independently confirmed by ellipsometry measurements of dried samples. This chemistry-driven activation strategy bypasses the need for complex biosensors and offers a general route to small-molecule metabolite targets that are difficult to address with antibody- or aptamer-based recognition.

## Graphical abstract

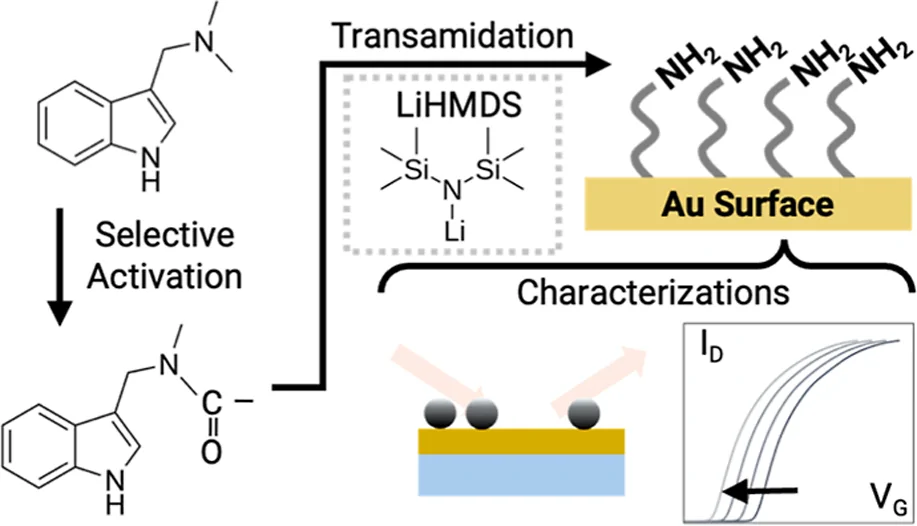

## Introduction

Among potent psychedelic drugs derived from plant metabolites, *N*,*N*-dimethyltryptamine (DMT) is a known endogenous tryptamine alkaloid, formed in mammalian tissue by N-methylation of tryptamine and detected in blood, urine, and cerebrospinal fluid of both healthy subjects and psychiatric patient cohorts, at basal levels as well as after exogenous administration.^1–9^ Its 2 *N*-methyl groups make the side chain a tertiary amine, and DMT acts on the central nervous system at trace concentrations.^4,5,10^ As known, DMT is a potent, short-acting psychedelic biomarker, producing marked changes in perception and mood that are now the subject of clinical trials in psychedelic-assisted detection and therapy.^5,11^ Interest in DMT accordingly spans neurochemistry, psychiatric diagnostics, and forensic screening. Endogenous brain levels range from 0.6 to 530 nM, so any platform intended for clinical or in vivo use must work within the nanomolar (1–100 nM) window.^8^ It is important to note that other tryptamine alkaloids carry the same tertiary amine side chain and therefore present the same measurement interference problem.^4,5^

The same functionality that confers this activity also makes the class difficult to measure at low concentration. The tertiary amine side chain offers no useful redox handle. Direct electrochemistry, therefore, lacks the selectivity and sensitivity needed at clinically meaningful concentrations, leaving these compounds effectively stealth analytes.^12^ The most sensitive DMT-specific platform reported to date, a molecularly imprinted polymer read out by electrogenerated chemiluminescence, covers 0.5 to 300 μM with a detection limit of 0.5–1.0 μM, which sits at the upper bound of the endogenous window and 3 orders of magnitude above its desired lower-end detection.^12^ On the other hand, liquid chromatography–tandem mass spectrometry (LC–MS/MS), which is the established reference method for endogenous tryptamines, reaches a very low level of 0.03 ng mL^–1^ (≈0.16 nM), and therefore covers the entire physiological window. However, it requires costly instrumentation, multistep sample preparation, and highly trained operators, which confines it to centralized medical facilities.^8,10^

Field-effect transistor (FET) sensors are an attractive sensing device alternative because they are label-free, potentiometric, and small enough to be used outside centralized facilities.^13,14^ After Bergveld introduced the ion-sensitive FET (ISFET) for neurophysiological measurements,^15,16^ the concept was extended to the extended-gate FET (EGFET), where the sensing membrane sits on a separate electrode wired to the gate of a conventional MOSFET,^14,17^ and to biologically sensitive FETs (BioFETs), in which the gate is coupled to enzymes, antibodies, nucleic acids, or cells.^18^ However, FET biosensing still faces two major limitations. First, the transistor itself responds to any change in interfacial charge and cannot distinguish one analyte from another. Selectivity must therefore be provided by a receptor on the gate, which restricts detection to targets for which a suitable receptor is available.^18^ Second, electrolyte ions screen the analyte charge over the Debye length (λ_D_), so binding events beyond λ_D_ of the gate can be largely screened.^13,19^ Strategies reported to overcome this screening limit include measurements in diluted electrolytes^19,20^ and poly­(ethylene glycol) layers that increase the effective screening length.^13,21^

In most FET biosensors, the receptor on the gate, such as an antibody, aptamer, or small-molecule receptor, determines both the selectivity and the distance between the binding event and the gate surface. Antibodies bind protein targets with high affinity and specificity,^22^ but their size (∼10 nm) can place much of the bound target outside λ_D_.^13^ Aptamers obtained by SELEX are smaller and have been used for small molecules such as cortisol and dopamine^23,24^ and for exosomes.^25^ For small molecules, the signal can originate from the target-induced rearrangement of the charged aptamer backbone near the gate, not from the charge of the target itself. This allows detection beyond the Debye limit,^26^ and target-bound immobilization further improves the sensitivity, even for uncharged cortisol.^27^ Small-molecule receptors are generally the most compact and can bring the binding site closer to the gate. Glycans have been used to detect influenza hemagglutinin at attomolar levels,^28^ phenylboronic acid to detect sialic acid on cell membranes^29^ and saccharides,^30^ and 4-mercaptobenzoic acid to detect histamine.^31^ In all of these cases, as with antibodies and antigens immobilized on a SAM or a linker layer,^22,32–35^ the selectivity is provided by the recognition group itself, and the monolayer serves only as a base layer. When these receptors are assembled into a sensor surface, most of them also require blocking and passivation steps, and each step can add variation in coverage and orientation.^36^ Antibodies and aptamers may also lose activity during storage and in biological media.^37,38^ In addition, each of these approaches requires a recognition group that binds the target selectively, and such groups are not readily available for chemically inert analytes such as tertiary amines.

Chemistry-driven sensing schemes can avoid the need for such a recognition element.^39,40^ Rather than screening a library for a binder and then building that binder into a stable, properly tailored surface, selectivity is designed into a predetection reaction that only the target functional group can undergo, so that the design variable becomes the choice of targeted reaction route rather than the outcome of a selection. This approach also eliminates the additional thickness of a biological recognition layer, which may help to keep the binding event within λ_D_.

To this end, we have recently shown that the selectivity problem can be addressed chemically for the model tertiary amine analyte, gramine.^39^ Selective N-oxidation and Polonovski rearrangement convert it to gramine amide, a surface-reactive derivative that binds covalently to amine-terminated SAMs.^39^ Building on this chemistry, here we couple it to an EGFET transducer in a three-step sensing strategy. In Step 1, the target amine is activated by that same N-oxidation/Polonovski sequence, introducing a reactive handle absent from the native molecule. In Step 2, the activated analyte is captured by an amino-terminated SAM on the FET gate electrode. In Step 3, the resulting shift in gate surface potential is transduced into a measurable electrical signal. Unlike the recognition groups described above, the terminal amine of the SAM acts only as a covalent anchor, and the selectivity is determined by the activation chemistry in solution. Since the analyte is captured directly on the molecularly thick monolayer, the bound molecule is expected to lie largely within λ_D_.^19,20^ In addition, because capture takes place before readout, the ionic strength of the readout electrolyte can be chosen independently of the binding chemistry. The EGFET then reads out this capture while keeping the semiconductor away from the sample, which shields it from biofouling and allows the gate electrode to be functionalized independently.^14,17,35,41^

To our knowledge, this combination of selective activation surface chemistry with an EGFET sensory scheme has not previously been applied to activated stealth amine biomarker detection. In this approach, gramine was activated and purified, and the gramine amide was then tested with EGFET sensors and independently confirmed by surface-sensitive measurements. We demonstrated that this approach achieves a very low limit of detection of 7 nM, well within the relevant physiological range reported for endogenous tertiary amines. The same approach can offer a route to diverse small-molecule biomarker targets that are difficult to address with antibody- or aptamer-based recognition.

## Results and Discussion

### Design and Operating Principle of the SAM-Gated EGFET

Figure 1 outlines the general platform design exploited in this study for gramine, used here as a structural surrogate for DMT: chemical activation of the analyte in solution (Step 1), capture at the gate electrode (Step 2), and electrical readout by the EGFET (Step 3). In this scheme, Step 1 addresses the absence of a redox handle by activating the analyte chemically before it reaches the electrode (Figure 1a, Approach 1). In this approach, gramine is first treated with hydrogen peroxide in acetone to yield gramine *N*-oxide, converting the tertiary nitrogen to a zwitterionic N^+^–O^–^ center while leaving the indole core untouched, as was demonstrated in our recent study.^39^

**Figure 1:**
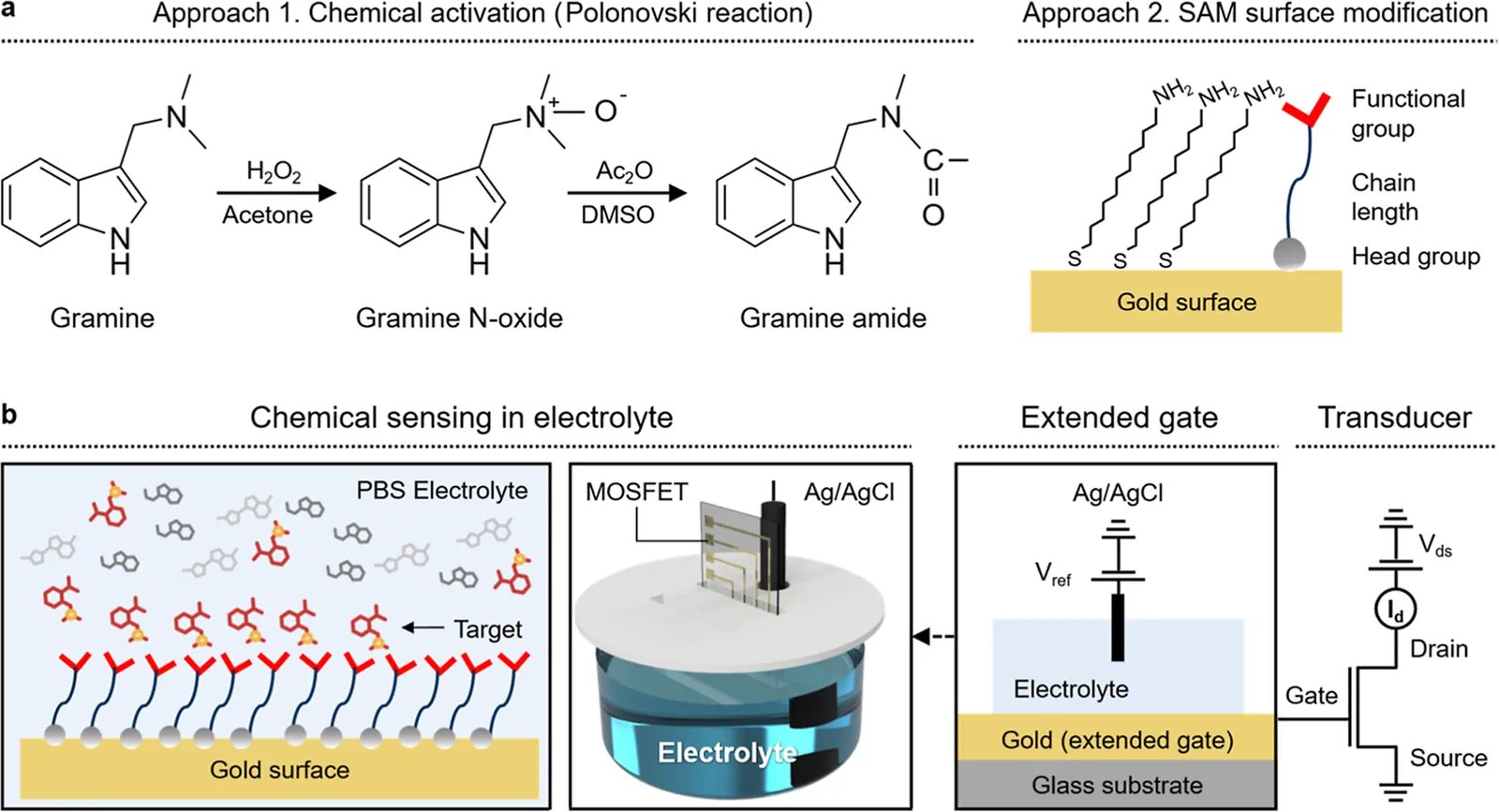
Schematic design of the chemically activated SAM-gated EGFET sensing platform. (a) Two complementary designs used to construct the sensing interface. In the chemical activation, gramine is oxidized to gramine *N*-oxide and subsequently converted to gramine amide through a Polonovski-type reaction. In the surface modification, an amino-terminated thiol self-assembled monolayer is formed on a gold surface, providing a reactive terminal amine interface for analyte immobilization. (b) Schematic illustration of the EGFET sensing configuration. Activated gramine amide is immobilized onto the SAM-modified gold extended gate in electrolyte, and the resulting surface-potential change is transduced through a MOSFET connected to the extended gate and an Ag/AgCl reference electrode.

Subsequent exposure to acetic anhydride in DMSO drives a Polonovski-type rearrangement that delivers gramine amide, in which the nitrogen now bears an electrophilic carbonyl.^42^ This sequence installs a reactive handle absent from the parent molecule and is the sole origin of chemical selectivity in the platform: N-oxidation proceeds only at tertiary amines, and the rearrangement proceeds only from the *N*-oxide, so primary and secondary amines and other endogenous nitrogen-containing species pass through both stages unchanged.

For this purpose, a functional SAM of 11-amino-1-undecanethiol is assembled on gold according to the known procedure.^32^

An alkanethiol monolayer presents three separately tunable structural elements: a thiol headgroup that anchors the molecule to the surface, an alkyl chain whose length sets film order, packing density, and dielectric thickness, and a terminal functional group that determines what the surface will react with. The amino (–NH_2_) terminus makes the surface complementary to the activated amide. The 11-carbon alkyl chain in thiols answers a separate requirement for active surface and electrode substrate. Indeed, our earlier demonstration of this chemistry used a 6-carbon analogue, which suffices when the film has only to be characterized; here the same film must also serve as a gate. Alkanethiols of eight carbons and fewer form conformationally disordered, liquid-like monolayers, whereas chains of ten and above pack into crystalline-like layers of markedly lower defect density.^32^

On a gate electrode, this difference in packing quality of organic monolayers is critical, because each pinhole in the monolayer constitutes a direct gold–electrolyte contact that leaks current and pins the local surface potential, and the resulting baseline drift is indistinguishable from a genuine analyte response. The chain length is therefore set by the transducer, not by the coupling chemistry, which proceeds equally well on the 6-carbon analogue.^39^ Indeed, in the presence of lithium hexamethyldisilazide (LiHMDS) as a strong base and nucleophile, the surface-bound primary amine attacks the amide carbonyl carbon.^43^ The resulting selective N–C bond cleavage at the interface forms a new N–C­(O)–gramine bond, covalently immobilizing the analyte on the surface by transition-metal-free transamidation.^43^

Finally, the electrical readout is provided by the EGFET sensing transistor (Figure 1b).^16,17^ In this design, the SAM-modified gold film serves as the extended selective gate: a separate electrode immersed in 0.1× PBS electrolyte and wired to the gate terminal of a commercial MOSFET, which itself remains outside the solution. This separation of detecting electrical elements is an important practical advantage of the EGFET geometry, since only an inexpensive, disposable gold-on-glass chip contacts the biological matrix. The semiconductor stage is shielded from ionic ingress and biofouling and can be reused across measurements.

An Ag/AgCl reference electrode in the same electrolyte supplies the gate bias (*V*_ref_), while the drain current (*I*_d_) is monitored at constant drain–source bias (*V*_ds_). This efficient readout is possible because the reference electrode fixes the potential of the bulk electrolyte. The effective gate bias detected by the MOSFET channel is the applied *V*_ref_ offset by the interfacial potential drop at the gate/electrolyte boundary.^16^

In this sensory design, any change in the charge, resistivity, or dipole distribution at that functionalized gate interface, therefore, registers as a corresponding shift in the threshold voltage of the transfer characteristic.^44,45^ Transamidation covalently immobilizes the activated analyte on the SAM-modified gate, and the resulting change in interfacial composition and adsorbed layer thickness alters the surface potential and thereby shifts the threshold voltage.

### Chemical Activation of the Tertiary Amine

Initially, Step 1 was verified on the isolated product before the activated analyte was brought to the electrode (Figure 2). FTIR identifies the functional group that the activation installs (Figure 2a). The gramine amide spectrum displays a characteristic carbonyl C=O absorption at approximately 1650 cm^–1^ (dotted line), which is absent from the gramine starting material.^39,46^ We reported previously that this band is also absent from the gramine *N*-oxide intermediate, so its appearance in the final product alone marks the installation of the carbonyl group.

**Figure 2:**
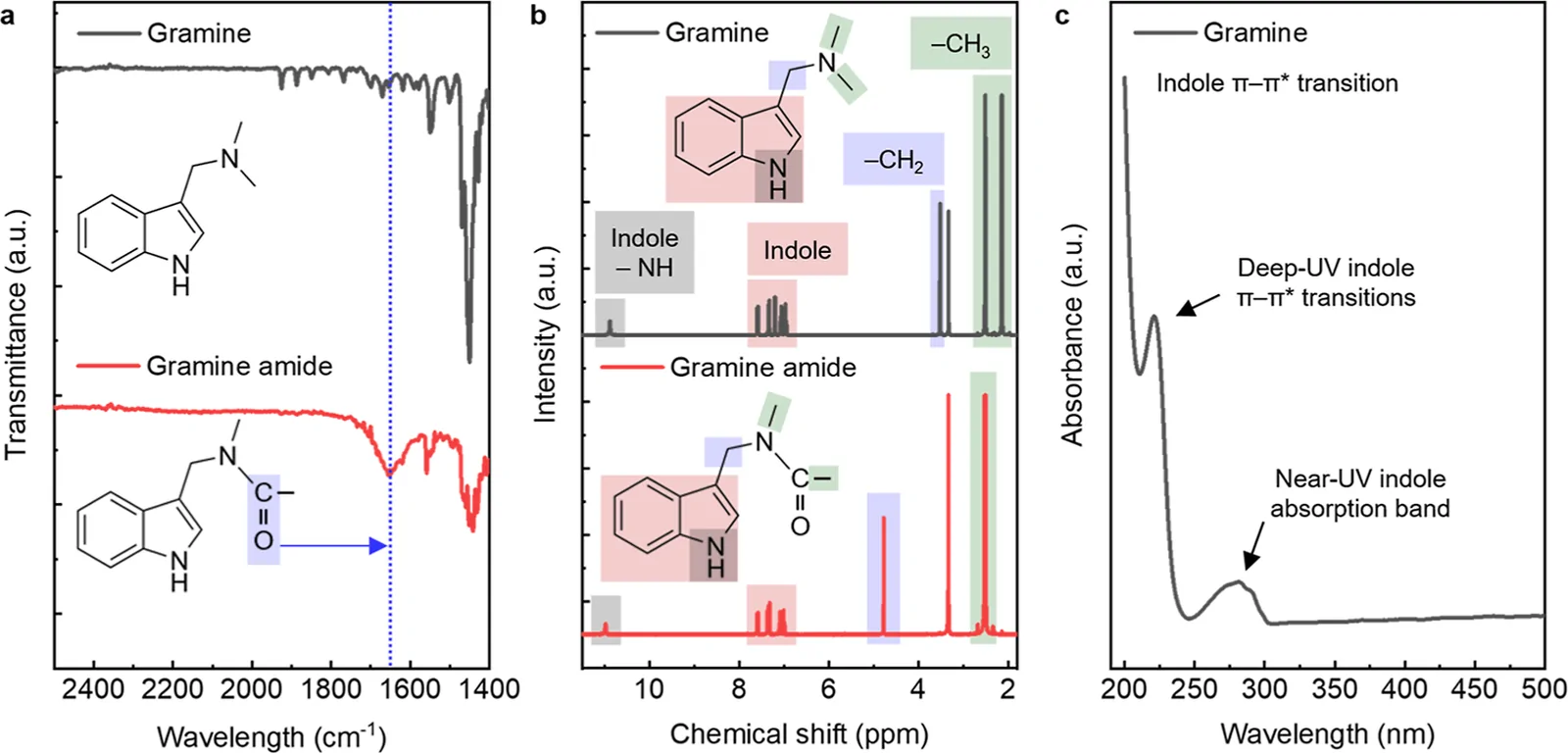
Chemical characterization of gramine activation to gramine amide. (a) FTIR spectra of gramine and gramine amide showing the emergence of an amide carbonyl absorption after chemical activation. (b) NMR spectra of gramine and gramine amide confirming changes in the chemical environment of the side-chain methyl and methylene protons while preserving the indole proton signals. (c) UV–vis absorption spectrum showing characteristic indole-derived deep-UV π–π* transitions and a near-UV indole absorption band.

Next, ^1^H NMR locates the transformation within the molecule (Figure 2b). In gramine, the N-bound –CH_3_ protons appear at approximately 2 to 3 ppm and the indole-bound –CH_2_ protons at 3.5 ppm, the shifts expected for a neutral tertiary amine.^47^ In the gramine amide spectrum, that resonance moves downfield to near 4.8 ppm, the shift expected once an electron-withdrawing carbonyl is bound to the nitrogen.

This –CH_2_ signal appears as a single peak rather than the diastereotopic pair we reported for the *N*-oxide intermediate, whose nitrogen is a stereogenic center.^39,48^ The indole aromatic protons between 7 and 8 ppm are unchanged across both spectra, so the reaction is confined to the side-chain tertiary amine and the indole core remains structurally intact.^39^ Read together with the carbonyl band in Figure 2a, these shifts establish that the amide formed at the side-chain nitrogen. Finally, UV–vis absorption provides a complementary view of changes in electronic conjugation (Figure 2c). As observed, gramine absorbs strongly below 230 nm and shows a weaker, broader band centered near 280 nm, the deep-UV and near-UV π–π* transitions characteristic of the indole chromophore.^49,50^

The two activation steps differ considerably in yield. Because the zwitterionic *N*-oxide is much less soluble in acetone than gramine, it precipitates as it forms and is isolated in ≈95% yield, while unreacted gramine remains largely in solution. The Polonovski step gives gramine amide in ≈50% isolated yield. Unreacted *N*-oxide is removed by liquid–liquid extraction (see Experimental Methods). The ^1^H NMR spectrum of the isolated gramine amide (Figure 2b) shows neither the side-chain resonances of gramine nor the diastereotopic –CH_2_ doublets of the *N*-oxide at 4.43 and 4.75 ppm,^39^ and the FTIR spectrum shows the amide carbonyl band (Figure 2a). Thus, no remaining starting material or intermediates were detected in the product used for sensing. For real biological samples, the activation would be performed on the sample itself, and the conversion efficiency of the activation sequence would therefore need to be included in the calibration, for example by treating standards with the same procedure.

The selectivity of this activation sequence was established in our earlier work.^39^ Tryptamine, an indole-based primary amine, showed none of the diagnostic downfield shifts when treated under identical oxidation conditions, and it also produced no appreciable rise in solution conductivity, which increases when the zwitterionic N^+^–O^–^ center of the *N*-oxide forms. Because zwitterions carry no net charge and are not expected to contribute to the ionic strength,^51^ this increase more likely arises indirectly: the large dipole moment of the *N* ^+^–O^–^ center may enhance the solvation and dissociation of background electrolytes in methanol, thereby increasing the number of mobile ions.^39^ Gramine, in contrast, exhibited both signatures, with a 4.6-fold increase in relative solution conductivity and a −5.7 mV shift in ζ-potential upon *N*-oxide formation.^39^ Neither primary nor secondary amines form *N*-oxides under these conditions, so the rearrangement, which proceeds only from the *N*-oxide, cannot be initiated on them.^39,52^ The activation step therefore acts as a chemical filter on the analyte before it reaches the gate.

This mode of filtering differs from the way selectivity is normally obtained in a biosensor, and the difference is where the significance of the design lies. Recognition is built by synthesis rather than selected from a library: step 1 is a reaction that only the target functional group can undergo, so a molecule lacking that group yields no product at all rather than a weaker one.^40^ Because the discriminating event is a reaction, it can be specified in advance and verified directly, as the spectra above establish for gramine. As known, an aptamer takes its specificity from whatever a SELEX selection returns, and an antibody from whatever an immunization and screening round produces. In both cases, the specificity is fixed once the binder exists, and it can be assessed only afterward, by testing the binder against a panel of analogs.^33,34,53,54^

Our approach, by contrast, is not tied to one compound in this way, and its reach is correspondingly wider for a whole class with similar functionalities. One activation sequence addresses every analyte carrying the reactive group, and further reaction steps can be designed into the sequence where finer discrimination is required. Selectivity becomes a design variable in this platform rather than a property that has to be searched for. For the target in our experiment, the product carries the electrophilic amide carbonyl required for Step 2, in which the surface-bound primary amine attacks that carbonyl and immobilizes the analyte on the SAM.

### Immobilization on the Amino-Terminated SAM

In this section, atomic force microscopy (AFM) imaging was used to monitor surface morphology changes after modification and adsorption (Figure 3a). Bare gold electrodes possess the grainy texture of an e-beam evaporated film, with 20–30 nm polycrystalline grains and an average microroughness (*R*_a_) of 1.12 ± 0.03 nm as measured over 500 nm × 500 nm surface areas. After the SAM forms, *R*_a_ falls significantly to 0.83 ± 0.03 nm, indicating smoothing of the gold electrode surface. This decrease is caused by the thiol molecules filling the grooves between the gold nanograins along with the grain tops.^55^

**Figure 3:**
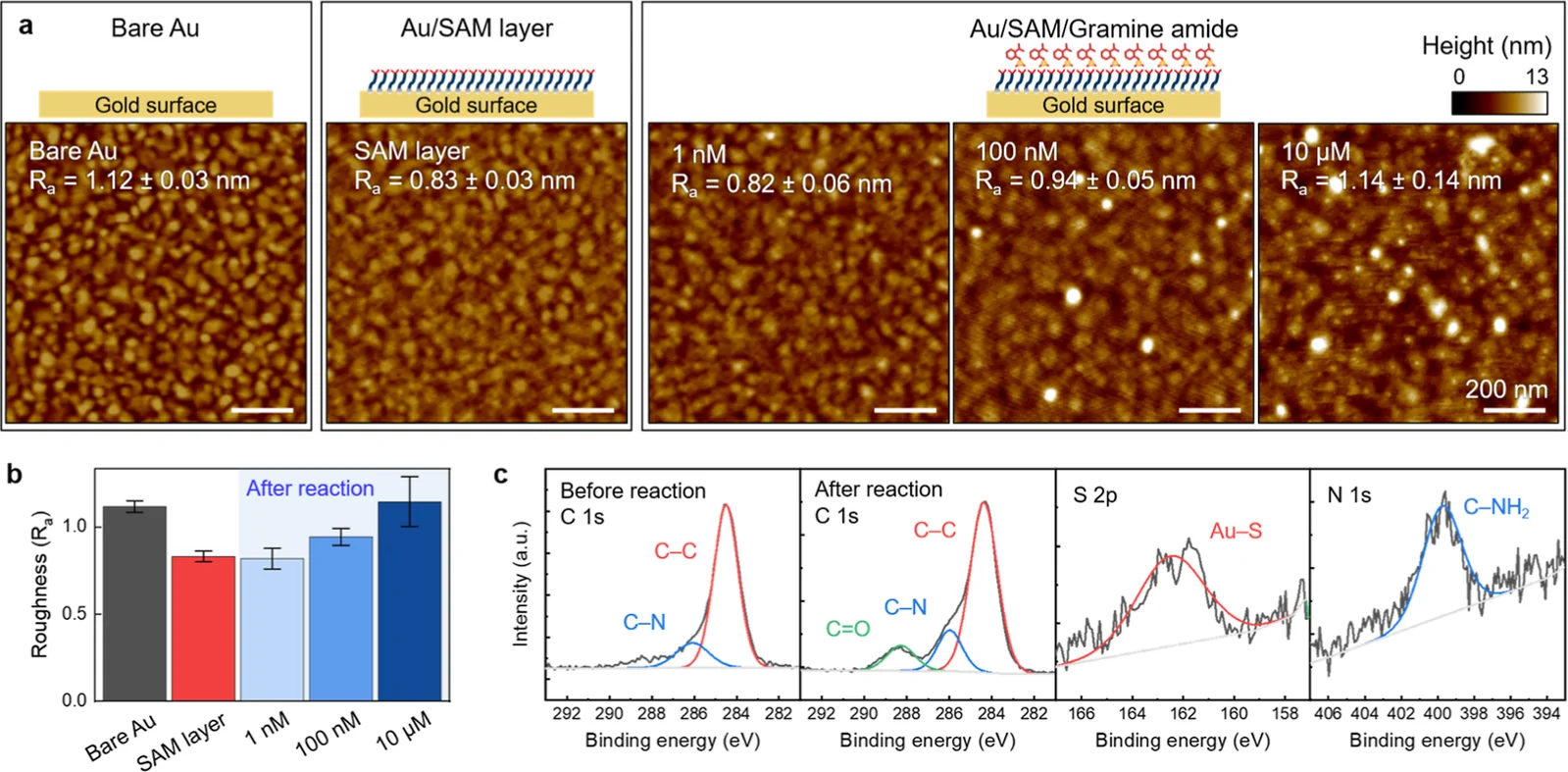
Surface morphology and chemical composition of the SAM-modified Au sensing layer after gramine amide immobilization. (a) AFM topography images of bare Au, Au/SAM, and Au/SAM/gramine amide surfaces prepared with different gramine amide concentrations. (b) Quantified surface roughness values showing concentration-dependent roughness increase after gramine amide immobilization. (c) XPS spectra showing C 1s chemical state changes before and after reaction, together with S 2p and N 1s spectra confirming Au–S bonding and primary amines on the surface.

This common smoothing effect is consistent with molecular assembly on reactive surfaces during SAM formation^56,57^ and is similar to that reported for the six-carbon SAM analogue.^39^ This points to a continuous layer with no large uncovered patches, which the sensory scheme requires for further potential monitoring.

Furthermore, at the lowest concentration of adsorbed biomarker tested in this study (1 nM), the SAM surface is indistinguishable from the pristine monolayer within experimental error. However, the microroughness then increases steadily as the concentration of biomarker increases, reaching a steady increase rate across the four-decade concentration range studied here (Figure 3a,b). At intermediate concentrations, discrete higher surface features begin to appear across the scanned area at approximately 100 nM, and the spread in roughness values across samples widens sharply due to larger-scale aggregation processes. A small shift in the average values paired with a much wider distribution is the signature of discrete adsorbates nucleating on an otherwise smooth template, rather than uniform conformal growth. Driven to completion, the same reaction roughly doubles the microroughness of a 6-carbon monolayer,^39^ whereas the surfaces examined here fall well short of that value even at the highest concentration tested. Neither LiHMDS alone nor nonactivated gramine produced any appreciable change in microroughness.^39^ The surface probed here therefore operates well below saturation, the regime in which a calibrated, concentration-dependent response is possible.

In addition, X-ray photoelectron spectroscopy (XPS) identifies the chemical bonding within the surface layer (Figure 3c). Before the reaction, the C 1s signal of the monolayer separates into C–C and C–N components, as expected for 11-amino-1-undecanethiol, which ends in a primary amine. After the reaction, a third component appears at higher binding energy and is assigned to C=O.^58,59^ This is the same carbonyl constituent that FTIR found in solution (Figure 2a), now present on the electrode. It marks the bond formed when the surface amine attacks the carbonyl of the activated analyte. The surfaces were rinsed carefully with absolute ethanol before measurement, so this carbonyl cannot come from loosely attached gramine amide left on the layer. Furthermore, the S 2p and N 1s regions confirm that the monolayer is anchored and terminated as intended. The S 2p peak near 162 eV belongs to gold-bound thiolate rather than to free thiol groups, so the layer is chemically bonded to the gold.^59^ The N 1s spectrum of the amine-terminated monolayer shows a single component near 400 eV assigned to the free primary amine.^60^

### Concentration and Time Dependencies of Adsorbed Layer Growth

For further confirmation of the amount of adsorbed material, spectroscopic ellipsometry was applied after specimen rinsing and drying (Figure 4).^39,61^ The effective surface layer thicknesses after exposure to different concentrations were obtained by fitting a glass/Au/organic stack at three angles of incidence, with the substrate and gold parameters held fixed and only the organic overlayer allowed to vary (Figure S1). Each value is the mean of five positions on the sample (see Experimental Methods). Figure 4a reports the total stack thickness at each stage of preparation. The gold film was determined to be 110 ± 1 nm. Assembly of 11-amino-1-undecanethiol brings the total stack thickness to 111.46 nm, indicating an added monolayer thickness of 1.46 ± 0.15 nm. This value is close to the extended length of an 11-carbon alkanethiol standing on gold and to reported literature values, thus confirming the monolayer nature of bound thiols.^62^

**Figure 4:**
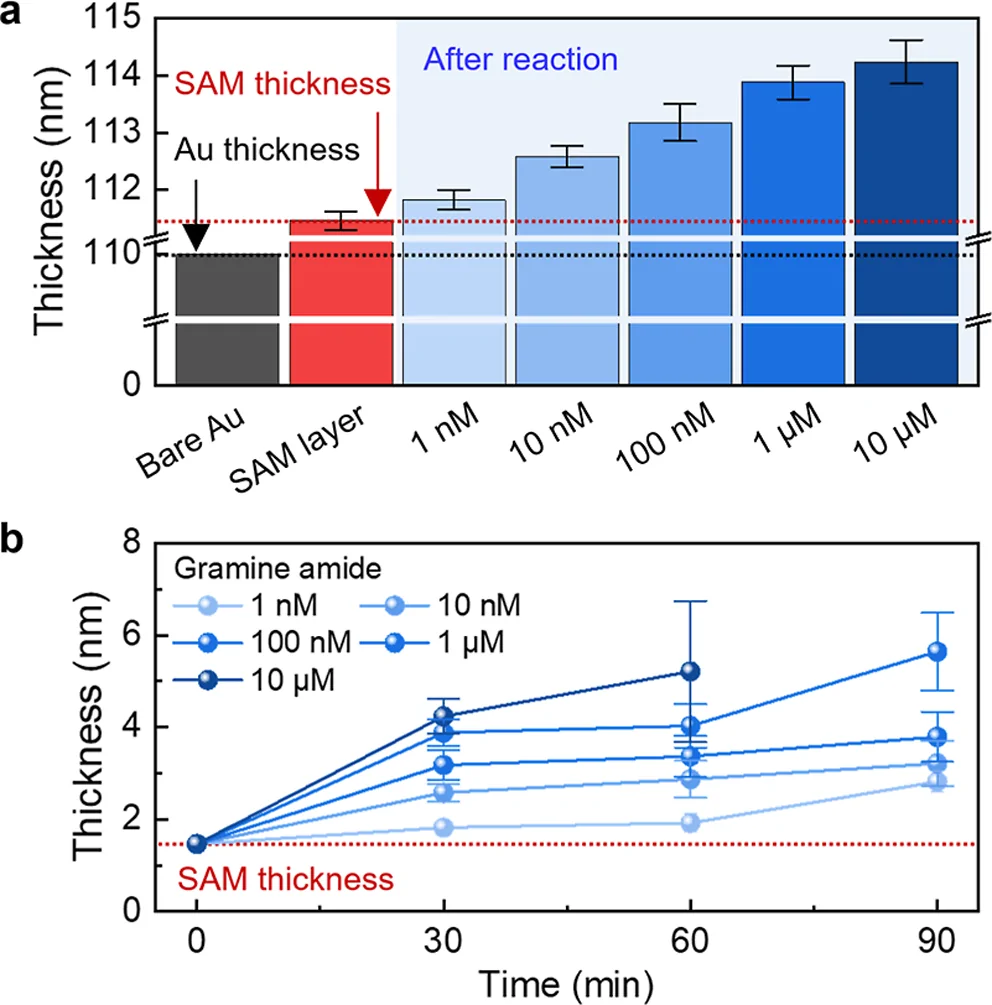
Ellipsometry analysis of layer growth during SAM formation and gramine amide immobilization. (a) Thickness changes measured after Au deposition, SAM formation, and gramine amide immobilization at different concentrations. (b) Time-dependent thickness evolution showing progressive gramine amide immobilization on the SAM-modified Au surface as a function of concentration and reaction time.

Next, reaction with the gramine amide for 30 min increases the effective thickness at every concentration tested, with the added overlayer growing steadily from 1 nM to 10 μM (Figure 4a). The thickness increment scales linearly with the logarithm of the analyte concentration (Figure 4a). A least-squares fit from 1 nM to 10 μM gives 0.61 nm per decade with *R* ^2^ = 0.99. A logarithmic rise is expected for a surface reaction that consumes a finite number of anchor sites of heterogeneous reactivity, as described by the Temkin isotherm, θ = (*RT*/*b*)·ln­(*K*_T_ *C*),^63,64^ rather than by a single-site Langmuir isotherm,^65^ for which the coverage goes from 10% to 90% within less than two decades of concentration before saturating. Growth is thus steepest at low coverage, as anticipated for progressive consumption of the surface amines. The lack of a plateau suggests that unreacted amine sites remain accessible at the highest concentrations. It is worth noting that this behavior facilitates the ability of surface-sensitive FET devices to cover several decades of concentration rather than a narrow window.^23,66^

The measured thicknesses can be converted into an analyte-to-anchor-site ratio using the grafting densities obtained from the ellipsometric data (Supplementary Note 1, Table S1). Assuming that the two organic layers pack at a comparable density, the coverage rises from 0.25 analyte per surface amine site at 1 nM to 1.91 at 10 μM. In this uniform-layer model, the ratio passes through unity between ≈40 nM (equal density) and ≈300 nM (70% density), that is, at around 100 nM. Because a ratio above unity cannot result from one-to-one covalent capture, we attribute the excess thickness above this crossover to physisorbed aggregates on top of the covalently bound layer. This is consistent with the topographical images, where discrete aggregates begin to appear at ≈100 nM (Figure 3a). However, the electrical response keeps increasing with no change in slope across this crossover (Figure 5c), which suggests that unreacted amine sites are still accessible after the aggregates form. The actual layer therefore likely differs from the uniform overlayer assumed in fitting the ellipsometric data (Figure S1). Because the aggregates are laterally patchy, a ratio above unity should be regarded as an average over thick aggregate islands and incompletely reacted regions, rather than as evidence that every anchor site is occupied.

**Figure 5:**
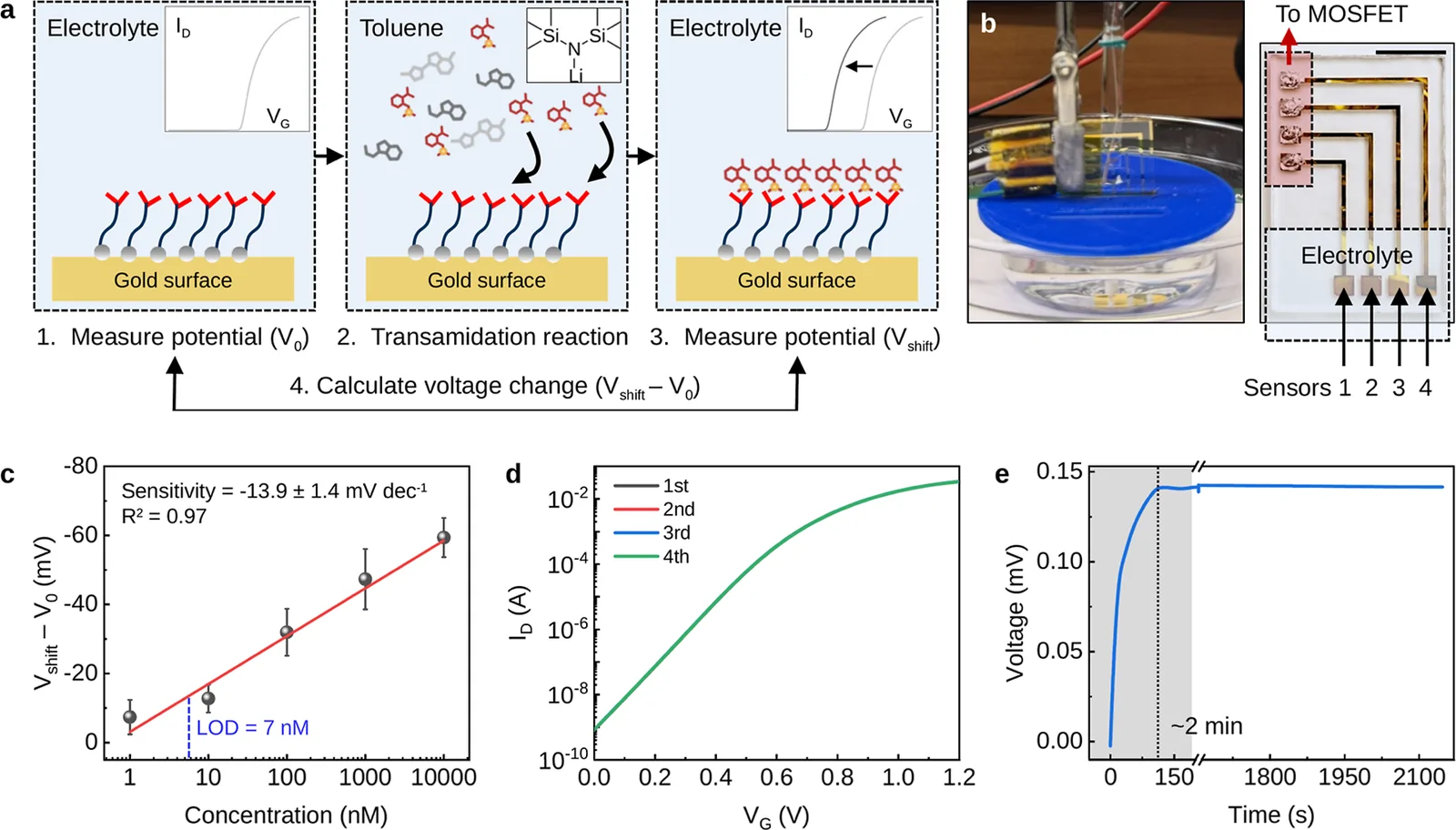
EGFET sensing mechanism and concentration-dependent electrical response. (a) Schematic of the EGFET sensing workflow. (b) Photograph and schematic of the EGFET measurement setup, including the Au extended-gate sensing electrodes, electrolyte, Ag/AgCl reference electrode, and MOSFET connection. (c) Calibration curve showing voltage shift as a function of gramine amide concentration, with a sensitivity of −13.9 ± 1.4 mV dec^–1^, *R* ^2^ = 0.97, and LOD = 7 nM. (d) Repeated transfer curves showing consistent transistor behavior over four sensing cycles. (e) Long-term time-dependent EGFET output.

The apparent coverage at the highest concentration is largely independent of the reaction conditions. In our earlier study, the same transamidation was carried out on a 6-carbon amino-thiol monolayer at 1 mM for 15 h, and the grafting densities obtained there, 4.7 molecules nm^–2^ for the monolayer and 10.3 molecules nm^–2^ for the amide layer, correspond to 2.2 amide molecules per thiol under the same equal-density assumption applied here (Supplementary Note 1).^39^ In the present work, an apparent ratio of 1.9 per anchor site is reached at 10 μM within 30 min, although the chain is five carbons longer and the solution is a hundredfold more dilute (Figure 4a).

The similar values obtained from these 2 quite different preparations suggest that the maximum apparent coverage is determined mainly by how densely the aggregated molecules pack on top of the covalently bound layer, rather than by the extent of covalent reaction. However, within the concentration range studied here, we found no indication of anchor-site saturation, as the electrical response remains log-linear up to 10 μM (Figure 5c). Above the crossover, the ellipsometric thickness contains a growing contribution from aggregates and should be regarded only as an apparent measure of covalent capture. Considering that endogenous DMT levels have been reported in the range of 0.6–530 nM,^8^ the concentration range examined here appears to cover most of the physiologically relevant window, and an extension above 10 μM is not expected to be necessary for the intended application.

At longer reaction times, the effective thickness increases further, and the increase is largest at the highest concentrations (10 μM at 60 min and 1 μM at 90 min, Figure 4b). Although the uniform-layer model predicts that the anchor sites are fully occupied at 1–10 μM after 30 min (Table S1), unreacted sites appear to remain, as discussed above. We therefore attribute the sharp increase in thickness mainly to growth of the surface aggregates (Figure 3a) and not to further covalent capture. Once aggregates have nucleated, the indole and amide groups of the captured molecules may retain additional gramine amide through π–π stacking between indole rings^67,68^ and hydrogen bonding between the indole N–H and the amide carbonyl.^69,70^ Unlike site-limited (Langmuir-type) adsorption,^65^ these adsorbate–adsorbate interactions are not limited by the number of surface sites, so the adsorbed amount does not reach a plateau, similar to BET-type multilayer adsorption.^64,71^ Aggregate growth requires a critical surface density of adsorbed molecules for initial nucleation and may then accelerate sharply with concentration, which is consistent with its becoming significant only at long reaction times and most pronounced at the highest concentrations. These two data points also have large standard deviations (±1.5 and ±0.9 nm, compared with ≤0.5 nm for the other points), which is consistent with uneven island growth. For island-like layers, the uniform-overlayer model gives an area-averaged effective thickness. For this reason, all sensing measurements were made after a 30 min reaction.

To confirm that the thickness increase originates from the activated analyte, the SAM was also exposed to gramine and gramine N-oxide under the same coupling conditions for 90 min at the highest concentration used in this study (10 μM; a saturated solution for the poorly soluble N-oxide). Neither control changed the thickness beyond the standard deviation of the pristine SAM, whereas gramine amide clearly increased the thickness after the same reaction time, even at 1 nM (Figure S2). This result is consistent with the absence of the carbonyl group required for transamidation in both compounds. The absence of a measurable physisorbed layer also suggests that the aggregates formed by gramine amide at high concentrations grow mainly on a primary layer.

### Electrical Response of the SAM-Gated EGFET

The final step converts the capture event into an electrical signal that an FET sensory system can monitor (Figure 5). The experimental procedure includes several stages. The SAM-modified extended gate is first immersed in 0.1× PBS electrolyte, and its baseline transfer characteristic is recorded to obtain the value of *V*_0_. The gate is then withdrawn and exposed to the transamidation reaction in anhydrous toluene, in which the activated gramine amide and LiHMDS effect the surface capture described above. After rinsing to remove unreacted reagent and physisorbed material, the gate is returned to fresh electrolyte and remeasured to obtain *V*_shift_. The difference *V*_shift_ – *V*_0_ is taken as the sensor response. The negative shift may be attributed to the accumulation of the indole moiety and the amide dipole at the gate surface, which changes the interfacial potential and thus lowers the gate bias needed to reach a given drain current (Figure 5c).

4 gold sensing pads are patterned side by side on a single chip, each serving as an independent extended gate and connected by a separate trace to the measurement circuit (Figure 5b). This multiplexed geometry allows several gates to be prepared under identical conditions and read sequentially, so that a single fabrication run provides replicate measurements and an on-chip reference gate for common-mode correction. In operation, the pads are immersed in the electrolyte together with an Ag/AgCl reference electrode and connected off-chip to the MOSFET stage.

As measured for different concentrations, *V*_shift_ – *V*_0_ becomes more negative with increasing gramine amide concentration, with a sensitivity of −13.9 ± 1.4 mV per decade and *R* ^2^ = 0.97 across four decades from 1 nM to 10 μM (Figure 5c).

To better understand the adsorption mechanism underlying this calibration, we compared the electrical response with the ellipsometric thickness increment measured under the same reaction conditions (30 min). Both quantities increase linearly with log *C* over four decades without reaching a plateau (Figure S3). This behavior is not well described by a single-site Langmuir isotherm. This model assumes identical, independent sites and therefore predicts saturation within about two decades of concentration.^64,65^ Dose curves of other transistor biosensors with SAM-functionalized gold gates have likewise been described with coverage-dependent isotherms such as the Frumkin isotherm.^72^ The Temkin isotherm, θ = (*RT*/*b*)·ln­(*K*_T_ *C*), accounts for this by assuming that the adsorption energy decreases linearly with coverage because of surface heterogeneity and adsorbate–adsorbate interactions, so that the coverage becomes linear in log *C* over a wide range of coverage.^63,64^ Both of our data sets fit this isotherm well (*R* ^2^ = 0.99 for Δ*d* and 0.98 for *V*_shift_ – *V*_0_, Figure S3), and the Temkin constants obtained separately from the two measurements, *K*_T_ ≈ 5.5 × 10^9^ and 2.0 × 10^9^ M^–1^, agree within a factor of 3, suggesting a common origin. The difference is comparable to the fitting uncertainty (log *K*_T_ = 9.74 ± 0.20 and 9.29 ± 0.23). Since covalent capture is irreversible, we treat these values as apparent constants at the fixed reaction time of 30 min.

Such a distribution of site reactivity is physically plausible in our system. Amine termini on the polycrystalline gate differ in local packing, protonation state, and accessibility near grain boundaries, and captured molecules and growing aggregates increasingly block access to neighboring sites. The most reactive sites are therefore likely to be consumed first, and the effective affinity is expected to drop as coverage increases. The continued rise of the electrical response above the crossover is consistent with this picture and suggests that the anchor sites do not behave as a single population that saturates. Together with the analyte-to-site ratios in Table S1, these data suggest two regimes.

Below the crossover (≲0.1 μM), the analyte is captured at amine sites of varying reactivity across the polycrystalline gate. Above it, aggregates account for most of the further increase in ellipsometric thickness, while capture continues at the remaining amine sites. However, the two measurements should not be expected to match exactly. Δ*d* is measured on dried films in air and averages over the compact layer and the aggregates alike, whereas *V*_shift_ – *V*_0_ is measured in electrolyte, where the layer is hydrated, ionic screening limits the response to changes within λ_D_ of the gate, and a covalently bound molecule at the interface is expected to contribute more to the interfacial potential than a loosely packed aggregate farther away. The two quantities therefore follow the same trend but can be compared only semiquantitatively.

The intrinsic stability of the transducer was verified independently by repeated measurement cycles (Figure 5d). 4 successive gate-voltage sweeps were recorded on the same device under identical conditions, and the resulting transfer characteristics are indistinguishable on the scale of the figure.

Magnification of the subthreshold region is presented in Figure S4 in order to visualize fine shifts. We recorded that the forward and reverse branches of all 4 sweeps fall within a window of about 1 mV. Hysteresis in devices of this type originates predominantly from mobile ions in the electrolyte or from slow charge trapping in the gate stack, either of which would make the threshold voltage depend on the sweep history rather than on the surface composition.^73^ The observed 1 mV spread, which is below 10% of the smallest analyte-induced shift, is therefore negligible against the tens of millivolts generated by analyte capture, so the significant shifts shown in Figure 5c can be attributed to the increasing amount of the immobilized analyte.

Comparison of this 1 mV window with the calibration data identifies the origin of the detection limit. Repeated sweeps on a single gate agree to within about 1 mV, whereas the spread across the five independently fabricated gates measured at each concentration in Figure 5c ranges from 4.0 to 8.7 mV, on average 6× larger. The detection limit is therefore governed by device-to-device fabrication variability rather than by measurement noise, and it follows from the scatter in *V*_0_ between gates, to which the gold grain structure, the quality of the monolayer, and the reproducibility of the piranha pretreatment all contribute. The multiplexed chip layout offers a direct route to suppressing this contribution: since the four gates on a die share a single evaporation and a single monolayer immersion (Figure 5b), a gate withheld from the analyte reports the common-mode component of the scatter, which can then be subtracted from the remaining 3.

The temporal stability of the output is shown in Figure 5e. After the gate is returned to the electrolyte, the signal rises from zero and reaches a stable plateau within approximately 2 min, after which it remains constant for the remainder of the measurement, out to roughly 35 min. The 2 min interval represents the response time of the readout and is set by the equilibration of the electrical double layer at the gate/electrolyte interface rather than by the surface reaction, which is carried out beforehand. The extended plateau confirms long-term reading stability, so the recorded response is insensitive to the precise acquisition time within the measurement window.

The analysis of the published data shows high sensitivity of the sensory scheme suggested in this study (Table S2). Molecularly imprinted electrogenerated chemiluminescence, the most sensitive DMT-specific sensor reported to date, reaches 0.5–1.0 μM,^12^ 2 orders of magnitude above the limit of detection obtained here. Among extended gates for other small molecules, the ranking follows the recognition chemistry rather than the transducer: the closest architectural analogue, an aromatic thiol monolayer on gold that binds its analyte by reversible affinity rather than by covalent capture, resolves histamine only in the millimolar range.^31^ The device reported here reaches the nanomolar range on a disposable gold-on-glass chip read out by a commercial MOSFET, approaching the range accessible at centralized medical facilities with expensive LC–MS/MS^8,10^ (Table S2). The same comparison holds for a protein target on an almost identical platform: an extended gate formed from a carboxyl-terminated alkanethiol monolayer on evaporated gold and read out by a commercial MOSFET, as here, detects an antiviral antibody only after the capture antigen has been coupled by EDC/NHS activation and the surface has been blocked with ethanolamine, with measurements performed at modest concentrations in a diluted buffer.^33^

## Conclusion

In conclusion, we demonstrated a 3-step sensing strategy combining selective chemical activation, covalent capture on a self-assembled monolayer, and extended-gate field-effect transduction for the detection of electrochemically inert tertiary amine biomarkers such as gramine, a surrogate for the psychedelic biomarker DMT. Polonovski-type activation installs a reactive carbonyl absent from the parent molecule, which an amino-terminated monolayer on the gate then captures by transition-metal-free transamidation. This approach overcame the fundamental challenge of tertiary amine electrochemical inertness, enabling electrical detection of a biomarker that offers no useful redox handle. The device achieved a sensitivity of −13.9 ± 1.4 mV per decade and a limit of detection of 7 nM, within the reported endogenous range, while ellipsometry independently confirmed log-linear layer growth over the same concentration range. The trade-off is that the reactivity route is exercised in solution, during activation, rather than at the electrode, so the platform reports the total concentration of activatable tertiary amines.

Overall, this chemistry-driven sensing methodology, focused on designing selectivity into a reaction rather than selecting it from a binder library, established a versatile platform for converting chemically inert tertiary amines into surface-confined recognition events suitable for selective FET-based electronic biosensing with sensitivity in the nanomolar regime. The reactive activation and selective binding approach suggested here is broadly applicable to tertiary amine-containing biomarkers, a class of neurochemical and forensic significance, opening new opportunities for label-free detection of small-molecule biomarkers previously inaccessible through antibody- or aptamer-based recognition.

## Experimental Methods

### Materials

Gramine (≥97.5%), acetic anhydride (ACS reagent grade, ≥98.0%), 11-amino-1-undecanethiol hydrochloride, and lithium hexamethyldisilazide (LiHMDS, 1.0 M in toluene) were purchased from Sigma-Aldrich. Hydrogen peroxide (30% w/w aqueous solution) and acetone (HPLC grade) were obtained from Fisher Scientific. Anhydrous toluene, spectroscopic-grade dimethyl sulfoxide (DMSO), ethyl acetate, absolute ethanol, methanol, isopropyl alcohol, sulfuric acid, and 1× phosphate-buffered saline (PBS) were also sourced from Sigma-Aldrich. Deionized water was used throughout. All chemicals were used as received without additional purification. All reactions were carried out at ambient temperature. The 0.1× PBS used as the measurement electrolyte was prepared by 10-fold dilution of the 1× stock with deionized water.

### Chemical Activation of Tertiary Amines

Tertiary amines were activated as previously described.^39^ Substrates were converted to their corresponding amide derivatives by a 2-step sequence, and the starting material and the isolated product of each step were weighed on an analytical balance (MS304S, Mettler Toledo). The tertiary amine (1 mmol) was first dissolved in anhydrous acetone and treated dropwise with hydrogen peroxide (1.2 equiv, 30% w/w aqueous solution) at room temperature for 24 h to give the *N*-oxide intermediate. Gramine is much more soluble in acetone than the zwitterionic *N*-oxide, so the *N*-oxide precipitates as a white powder as it forms and is thereby largely separated from unreacted gramine. The crude product was recovered by rotary evaporation, collected by centrifugation, washed three times with a minimal volume of acetone to remove residual gramine, and dried under vacuum (isolated yield ≈95%). The *N*-oxide (1 mmol) was then dissolved in anhydrous DMSO under N_2_, acetic anhydride (1.2 equiv) was added in a single portion, and the mixture was stirred for 5 h at room temperature.

The reaction was quenched with deionized water, and gramine amide was separated by liquid–liquid extraction (solvent exchange) into ethyl acetate: unreacted zwitterionic *N*-oxide remains in the aqueous DMSO phase, whereas the neutral gramine amide partitions into the organic phase; the combined organic layers were washed 3× with deionized water to remove DMSO, acetic acid, and water-soluble byproducts, and the solvent was removed by rotary evaporation under reduced pressure. The extraction was repeated three times to maximize recovery, and the combined product was dried under vacuum and weighed (isolated yield ≈50%). The identity and purity of the *N*-oxide and gramine amide were confirmed by ^1^H NMR and FTIR (Figure 2).^39^

### Fabrication of the Functionalized Gold Sensing Layer

Glass substrates were cleaned with deionized water and dish soap, then rinsed sequentially with acetone, methanol, and isopropyl alcohol. Gold electrodes (100 nm thick) with a titanium adhesion layer (10 nm) were deposited on the glass substrates by e-beam evaporation through a shadow mask. Prior to functionalization, the gold surfaces were treated with piranha solution for 10 min. Caution: piranha solution reacts violently with organic material and must be handled with appropriate protective equipment. Monolayer formation was carried out under N_2_ in a Labstar Pro glovebox (MBRAUN) with H_2_O and O_2_ both below 1 ppm. Substrates were submerged in an ethanolic solution of 11-amino-1-undecanethiol (500 μM) for approximately 18 h to form a SAM presenting terminal primary amine (–NH_2_) groups. The functionalized substrates were then rinsed at least three times with ethanol and deionized water, sonicated for 3 min in fresh ethanol, and dried under a stream of N_2_.

### Transamidation Coupling to the SAM-Modified Surface

The isolated gramine amide was weighed and dissolved in anhydrous toluene to prepare a 1 mM stock solution (*M* = 202.3 g mol^–1^), from which working solutions from 1 nM to 10 μM were prepared by serial 10-fold dilution in anhydrous toluene immediately before use. Freshly prepared SAM-modified gold substrates were immersed in the solution under continuous stirring. LiHMDS was then added from a 1.0 M stock in toluene to a fixed final concentration of 10 μM in all experiments, independent of the gramine amide concentration. Reactions were run at room temperature for 30 min unless otherwise stated; the time-resolved series in Figure 4b used reaction times of 30, 60, and 90 min. All concentration-series data in Figures 3, 4a, and 5 were collected after 30 min. On completion, substrates were rinsed 3× with toluene to remove unreacted reagent and physisorbed material, rinsed with absolute ethanol, and dried under a stream of N_2_. Control experiments were carried out with gramine and gramine N-oxide under the same coupling conditions (10 μM, 90 min). Because the N-oxide is poorly soluble in toluene, it was dispersed in anhydrous toluene at a nominal concentration of 10 μM and centrifuged (6000 rpm, 5 min), and the saturated supernatant was used.

### Solution-Phase Characterization

^1^H NMR spectra were acquired at room temperature on a Bruker AV3 400 MHz spectrometer. Samples were dissolved in deuterated DMSO (DMSO-*d*_6_) at 0.1 mg mL^–1^, and at least 32 scans were accumulated per spectrum. Spectra were processed in Bruker TopSpin and MestReNova. Infrared spectra were recorded on a Bruker Vertex 70 spectrometer fitted with an attenuated total reflectance (ATR) accessory. Solutions of the purified products were drop-cast onto the ATR crystal and dried under vacuum for 24 h to remove residual solvent. Spectra were collected from 4000 to 1200 cm^–1^ as an average of 64 scans, with a background recorded before each sample and subtracted automatically.

### Surface Characterization

Surface morphology was examined with a Bruker Dimension Icon atomic force microscope operated in PeakForce Tapping mode according to the common procedures described previously.^74,75^ Samples were rinsed with toluene and ethanol and dried under vacuum before imaging. RTESPA-300 probes were used (nominal tip radius 8 nm, spring constant 40 N m^–1^, resonance frequency ∼300 kHz), with 512 to 1024 samples per line and scan rates of 0.5 to 1 Hz. Scans were collected over 3.0 μm × 3.0 μm regions, and microroughness (*R*_a_) was averaged over at least three 500 nm × 500 nm areas selected to avoid accumulated aggregates. Image flattening, height analysis, and roughness calculations were performed in NanoScope Analysis 2.0.

XPS spectra were collected on a Thermo Scientific K-Alpha spectrometer. Samples were dried under vacuum for at least 3 h before analysis. Survey spectra were acquired twice over 0 to 1350 eV in 1 eV increments, and high-resolution spectra were accumulated over 10 scans per element in 0.1 eV steps. All spectra were calibrated to the C 1s peak at 284.8 eV and analyzed in Thermo Scientific Avantage software.

Spectroscopic ellipsometry was performed on a Woollam M-2000U instrument at incidence angles of 65, 70, and 75°. Thicknesses were extracted in VWASE software using a multilayer model of the glass substrate, gold layer, and organic overlayer under ambient conditions. During fitting, the optical constants of the substrate and gold layer were held fixed, and only the parameters of the organic overlayer were varied. Each reported thickness is the mean of measurements at 5 independent positions on the sample, with the standard deviation taken across those positions.

### EGFET Assembly and Electrical Measurement

The SAM-modified gold film served as the extended gate. Each chip carried 4 independently addressable gold sensing pads, wired by separate traces to the measurement circuit, allowing several gates to be prepared and read from a single fabrication run. The extended gate was connected to the gate terminal of a commercial n-channel MOSFET (FDV301N, ON Semiconductor/onsemi, Scottsdale, AZ), which remained outside the electrolyte. Measurements were made in 0.1× PBS with an Ag/AgCl reference electrode (Sigma-Aldrich) supplying the gate bias. Transfer characteristics were recorded by sweeping the gate voltage from 0 to 1.2 V at a constant drain-source bias of 0.5 V using a Keithley 4200-SCS semiconductor characterization system, with drain current recorded on a logarithmic scale.

For each sensing experiment, a baseline transfer curve was recorded in fresh 0.1× PBS to give *V*_0_. The gate was then removed from the electrolyte, subjected to the transamidation coupling described above, rinsed, and returned to fresh 0.1× PBS, where a second transfer curve was recorded to give *V*_shift_. The reported sensor response is the difference *V*_shift_ – *V*_0_. Hysteresis was assessed by recording four successive transfer curves on the same device under identical conditions. Time-resolved output was recorded continuously for approximately 2100 s following immersion of the gate in electrolyte.

### Determination of the Limit of Detection and Statistical Analysis

The calibration curve was constructed by plotting *V*_shift_ – *V*_0_ against the logarithm of gramine amide concentration over 1 nM to 10 μM, and the sensitivity was taken as the slope of the least-squares fit. Ellipsometric thicknesses are reported as mean ± standard deviation over 5 positions per sample. Electrical measurements are reported as mean ± standard deviation over 5 independent devices per concentration.

## Supplementary Material



## Notes

Comparison of the actual surface morphology with the uniform-layer optical model used for ellipsometric fitting (Figure S1); control experiments for the transamidation coupling with gramine and gramine N-oxide (Figure S2); conversion of ellipsometric thickness to the analyte-to-anchor-site ratio (Supplementary Note 1, Table S1); comparison with reported DMT detection methods and EGFET sensors (Table S2); Temkin isotherm analysis of the ellipsometric and EGFET responses (Figure S3); transfer characteristics of the EGFET over four successive gate-voltage sweeps (Figure S4) (PDF)
