# Supplementary material for: Surface Reaction-Mediated Nanomolar Detection of Chemically Activated Plant Metabolite Biomarkers with Field Effect Sensors
Source: Langmuir. 2026 Oct 29;42(44):32960–71. doi: 10.1021/acs.langmuir.6c05228 (PMC13757961; doi:10.1021/acs.langmuir.6c05228)
Supplement: Supplementary file 1 [file la6c05228_si_001.pdf]

Supporting Information for:

Surface Reaction-Mediated Nanomolar Detection of Chemically Activated Plant Metabolite Biomarkers with Field Effect Sensors

Sehyun Park, Jinyoung Kim, Jinyoung Choi, Jaejun Lee, Vladimir V. Tsukruk *

School of Materials Science and Engineering, Georgia Institute of Technology, Atlanta, GA 30332, USA

* Corresponding author: vladimir@mse.gatech.edu

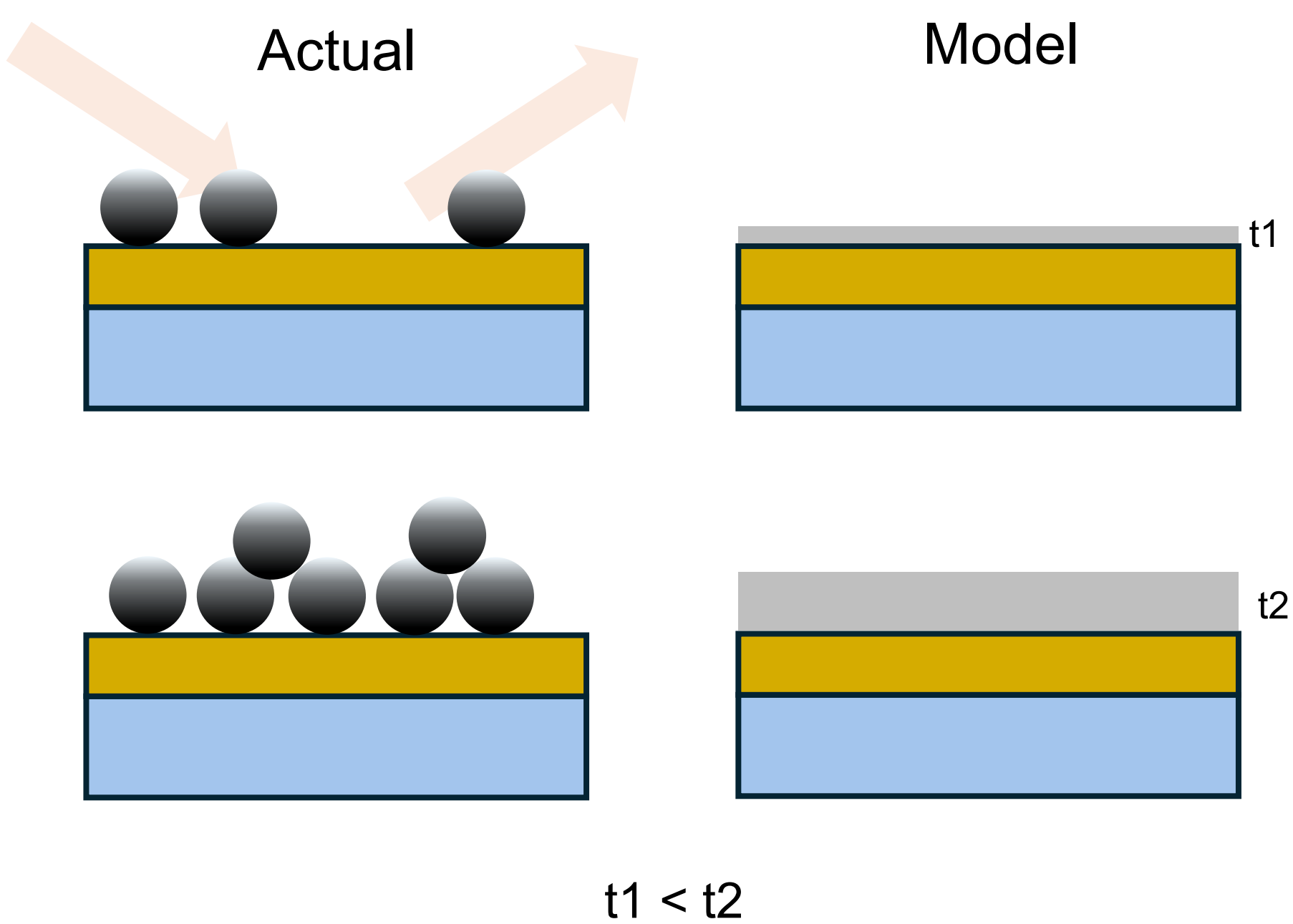

Figure S1. Actual surface morphology compared with the uniform-layer model used to fit the ellipsometry data. The reacted surface consists of an amino-terminated monolayer carrying covalently bound gramine amide, with additional material collected into discrete aggregates at higher concentrations and longer reaction times (left). The optical model treats this as a single continuous overlayer of uniform thickness (right). The fitted value is therefore an effective thickness averaged over the probe area, not the physical height of any individual feature.

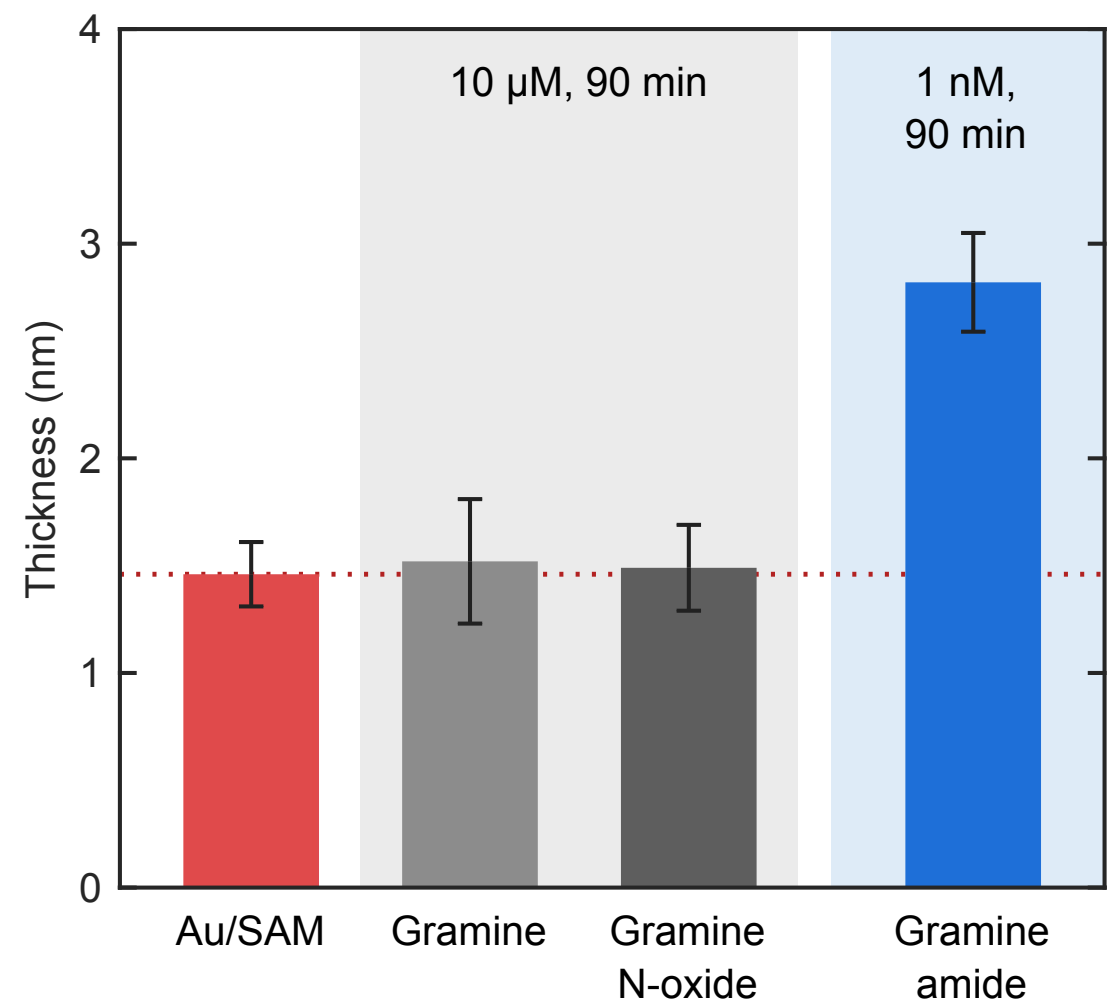

Figure S2. Control experiments for the transamidation coupling. Ellipsometric thickness of the pristine Au/SAM before reaction and of the SAM after reaction with gramine (10 µM) or a saturated solution of gramine N-oxide (nominal 10 µM) for 90 min, compared with gramine amide at 1 nM after 90 min. The dotted line marks the thickness of the pristine SAM.

Supplementary Note 1. Theoretical conversion of ellipsometric thickness to a molecular ratio.

For an organic layer of effective thickness d, mass density ρ, and molar mass M, the number of molecules per unit area (grafting density) is obtained from[1,2]

$$\sigma = \rho \cdot d \cdot N_A / M \tag{S1}$$

where N_A is Avogadro's number. $\rho \cdot d$ is the mass supported per unit area, and M/N_A is the mass of one molecule.

Applying this separately to the monolayer and to the layer added by reaction, and taking the ratio of the two,

$$\frac{\sigma_{added}}{\sigma_{SAM}} = \frac{\rho_{added} \cdot d_{added}}{\rho_{SAM} \cdot d_{SAM}} \times \frac{M_{thiol}}{M_{amide}} \tag{S2}$$

Taking the two organic layers to pack at a similar density, ρ cancels and the expression reduces to

$$\frac{\sigma_{added}}{\sigma_{SAM}} = \frac{d_{added}}{d_{SAM}} \times \frac{M_{thiol}}{M_{amide}} = 1.904 \times 1.0056$$
$$= 1.91\ (for\ the\ 10\ \mu M\ case) \tag{S3}$$

The ratio is fixed by the measured thicknesses and the two molar masses.

11-Amino-1-undecanethiol (203.39 g mol^{-1}) and gramine amide (202.26 g mol^{-1}) differ in molar mass by 0.6%, so the second factor is 1.0056 and the ratio is set almost entirely by the measured thicknesses.

Thicknesses are taken from the 30 min series in **Figure 4a** of the main text. The gold-coated substrate was fitted first and its thickness held fixed thereafter, so that each subsequent fit returns the total organic thickness above the gold. The monolayer measures d_{SAM} = 1.46 nm, and the layer added by the surface reaction is therefore $d_{added} = d_{total} - 1.46$ nm, where d_{total} is the total organic thickness measured after transamidation. At 10 µM, for example, d_{total} = 4.24 nm gives d_{added} = 2.78 nm (**Table S1**). Because a single uniform overlayer is assumed, d_{added} is an effective thickness averaged over the probe area rather than the physical height of an individual adsorbate (**Figure S1**).

A well-ordered monolayer is likely to be denser than an overlayer containing aggregates, so the equal-density values are upper bounds; the final column gives the corresponding lower bound at 70% of the monolayer density.[3] Interpolating unity coverage gives approximately 40 nM under the equal-density assumption and approximately 300 nM at the lower bound. Within that bracket the assignment is unambiguous at the ends of the series: 1 and 10 nM lie below one analyte per anchor site under either assumption, and 1 and 10 µM lie above it under either assumption. This bracket defines the upper limit of the sub-monolayer regime quoted in the main text. Because the uniform-layer model averages over laterally heterogeneous aggregates, a ratio above unity is a spatial

average and does not imply that every anchor site is occupied; the continued increase of the EGFET response above this bracket (Figure 5c of the main text) suggests that unreacted amine sites remain.

For reference, the same expression applied to the monolayer alone (d = 1.46 nm, M = 203.39 g mol^{-1}, ρ = 0.9 g cm^{-3})[4–6] gives 3.9 molecules nm^{-2}, somewhat below the 4.6 molecules nm^{-2} expected for a close-packed alkanethiolate lattice on Au(111).[7,8] This does not affect the ratio quoted above, which is independent of the density.

Table S1. Conversion of the ellipsometric thicknesses of **Figure 4a** into an analyte-to-anchor-site ratio. The total organic thickness is the fitted thickness above the gold; the added layer is that value less the 1.46 nm monolayer. The fourth column assumes that the added overlayer packs at the same density as the monolayer, which is an upper bound on the coverage, and the fifth assumes 70% of the monolayer density, which is the corresponding lower bound. Coverage crosses one analyte per anchor site between 10 and 100 nM under the equal-density assumption and between 100 nM and 1 µM at the lower bound. All values are from the 30 min reaction series.

Gramine amide	Total organic thickness (nm)	Added layer (nm)	Molecules per anchor site ($\rho_{added} = \rho_{SAM}$)	Molecules per anchor site ($\rho_{added} = 0.7\ \rho_{SAM}$)
1 nM	1.82	0.36	0.25	0.17
10 nM	2.58	1.12	0.77	0.54
100 nM	3.18	1.72	1.18	0.83
1 µM	3.88	2.42	1.67	1.17
10 µM	4.24	2.78	1.91	1.34

Table S2. Comparison of the present platform with reported methods for DMT and with extended-gate field-effect transistor (EGFET) sensors for targets of comparable size and functionality. The comparison is discussed in the main text.

Method	Target	Recognition element	Limit of detection	Ref.
EGFET with chemically activated analyte; disposable Au chip and commercial MOSFET (this work)	Gramine (DMT surrogate)	Amino-terminated C11 alkanethiol SAM, 1.5 nm; single covalent coupling step	7 nM (range 1 nM–10 µM)	This work
Electrogenerated chemiluminescence with molecularly imprinted polymer; optical readout	DMT	Imprinted polymer cavity; polymerization and template removal	0.5–1.0 µM (range 0.5–300 µM)	9
LC-MS/MS; benchtop instrument, centralized laboratory	DMT	None (chromatographic separation); multi-step sample preparation	0.03 ng mL^{-1} (≈0.16 nM)	10
EGFET	Histamine	Aromatic thiol SAM on Au; reversible affinity, no covalent capture	n/a (range 0.25–5 mM)	11
EGFET	Serotonin	Phenylboronic acid in a nanofilter film on Au	n/a (range 0.6–2 µM)	12
EGFET (organic)	(S)-Hyoscyamine (tropane alkaloid)	Molecularly imprinted polymer on Au; polymerization and template removal	1 µM (range 0–7 µM)	13
EGFET	Dopamine	Molecularly imprinted polymer on Au; polymerization and template removal	96 nM (range 40 nM–20 µM)	14
EGFET immunosensor	Anti-BHV-1 antibody (protein)	Carboxyl-terminated C16 alkanethiol SAM on Au; gE antigen (59 kDa) coupled by EDC/NHS, then ethanolamine blocking	Serum dilution < 2 × 10^{-3} (no molar limit quoted)	15

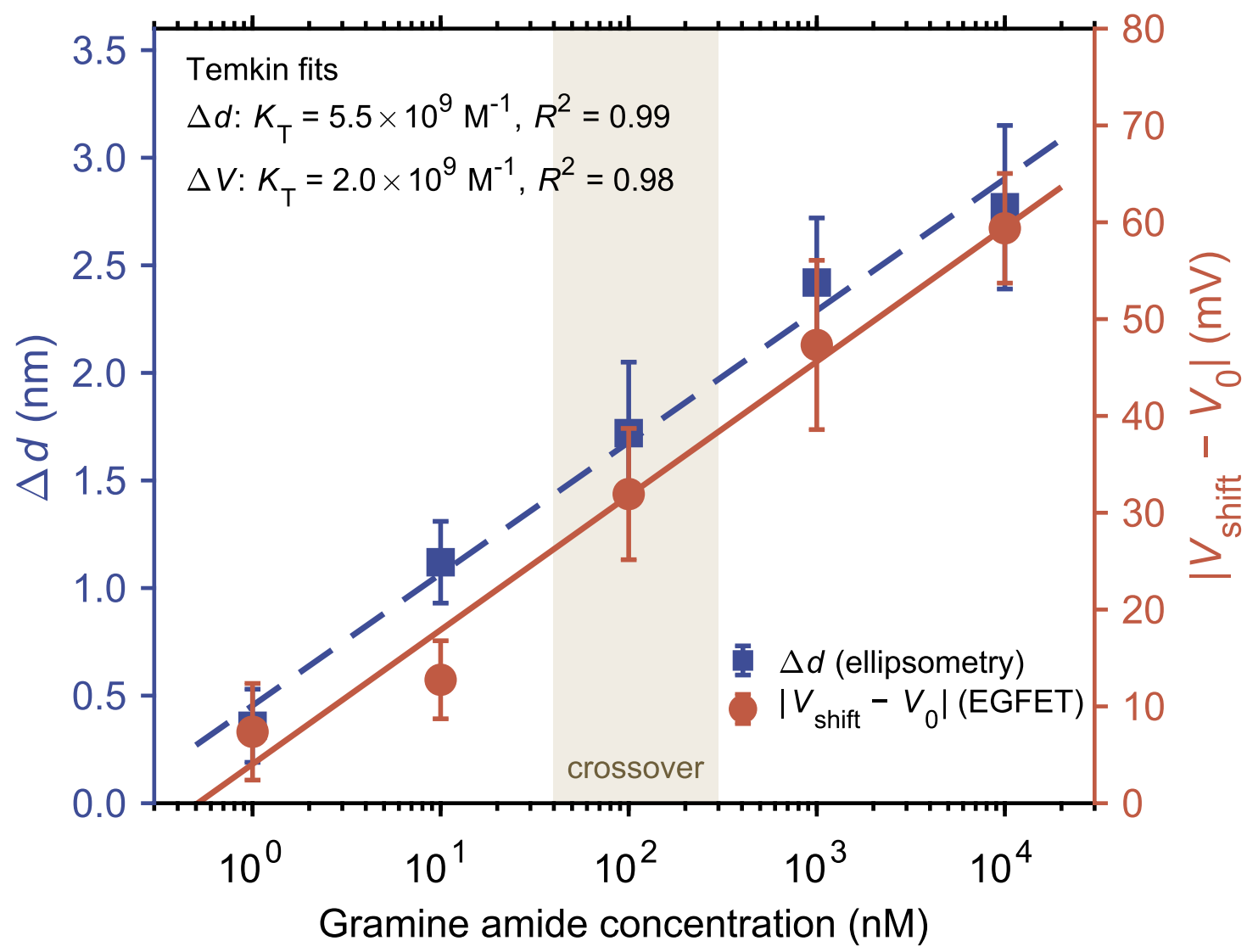

Figure S3. Temkin isotherm analysis of the ellipsometric thickness increment (Δd, squares, left axis) and the EGFET response ($|V_{shift} - V_0|$, circles, right axis) after a 30 min reaction at 1 nM–10 µM. Lines are linear fits to the Temkin isotherm, $y = B \ln(K_T C)$, yielding $K_T = 5.5 \times 10^9$ M^{-1} ($R^2 = 0.99$) for Δd and 2.0×10^9 M^{-1} ($R^2 = 0.98$) for the electrical response. The shaded band marks the crossover region (40–300 nM) in which the analyte-to-anchor-site ratio of the uniform-layer model reaches unity (Table S1).

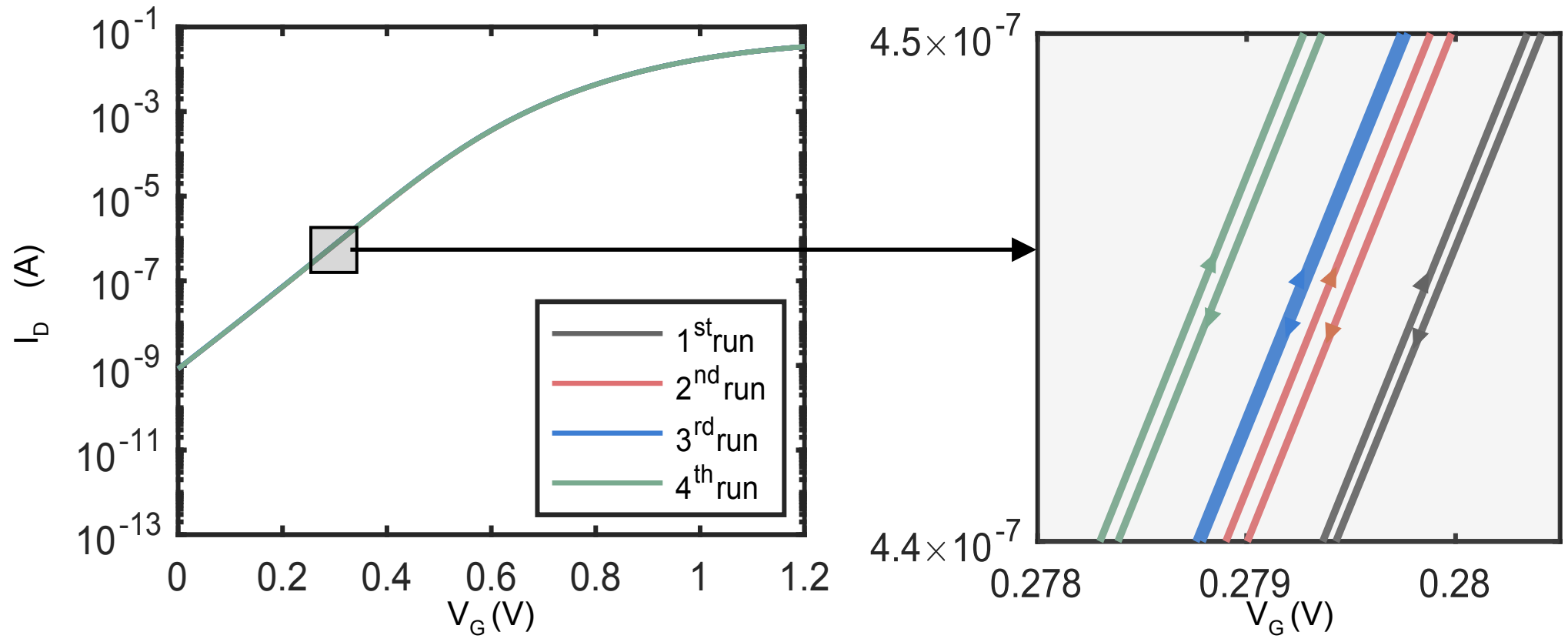

Figure S4. Transfer characteristics of the EGFET over four successive gate-voltage sweeps. (a) Drain current measured on four consecutive sweeps of the same device, plotted on a logarithmic scale. (b) Magnified view of the boxed region in (a); arrows indicate the sweep direction. The curves overlap closely on both scales, and the forward and reverse branches fall within approximately 1 mV, indicating that hysteresis from mobile ions or charge trapping in the gate stack is small compared with the analyte-induced shifts.

Supplementary References

(1) Bukharina, D.; Cauffiel, K.; Killingsworth, L. M.; Brackenridge, J. A.; Poliukhova, V.; Kim, M.; Brower, J.; Bernal-Chanchavac, J.; Stephanopoulos, N.; Tsukruk, V. V. Click-Chemistry-Enabled Functionalization of Cellulose Nanocrystals with Single-Stranded DNA for Directed Assembly. ACS Biomater. Sci. Eng. 2024, 10 (10), 6155–6166.

(2) Choi, J.; Yelipashev, M.; Poliukhova, V.; FitzPatrick, J.; Gogotsi, Y.; Lin, Z.; Tsukruk, V. V. Precision Grafting-From of Diblock Copolymer Brushes on MXene Nanosheets. Chem. Mater. 2025, 37 (20), 8238–8251.

(3) Aspnes, D. E. Optical Properties of Thin Films. Thin Solid Films 1982, 89 (3), 249–262.

(4) Techane, S.; Baer, D. R.; Castner, D. G. Simulation and Modeling of Self-Assembled Monolayers of Carboxylic Acid Thiols on Flat and Nanoparticle Gold Surfaces. Anal. Chem. 2011, 83 (17), 6704–6712.

(5) Hinterwirth, H.; Kappel, S.; Waitz, T.; Prohaska, T.; Lindner, W.; Lämmerhofer, M. Quantifying Thiol Ligand Density of Self-Assembled Monolayers on Gold Nanoparticles by Inductively Coupled Plasma–Mass Spectrometry. ACS Nano 2013, 7 (2), 1129–1136.

(6) Ahn, H.; Kim, M.; Sandman, D. J.; Whitten, J. E. Self-Assembled Monolayers of ω-(3-Thienyl)Alkanethiols on Gold. Langmuir 2003, 19 (13), 5303–5310.

(7) Love, J. C.; Estroff, L. A.; Kriebel, J. K.; Nuzzo, R. G.; Whitesides, G. M. Self-Assembled Monolayers of Thiolates on Metals as a Form of Nanotechnology. Chem. Rev. 2005, 105 (4), 1103–1170.

(8) Poirier, G. E.; Pylant, E. D. The Self-Assembly Mechanism of Alkanethiols on Au(111). Science 1996, 272 (5265), 1145–1148.

(9) Motchaalangaram, J. A.; Mahalingam, P.; Wallace, K. J.; Miao, W. Electrogenerated Chemiluminescence Coupled with Molecularly Imprinted Polymer for Sensitive and Selective Detection of N,N-Dimethyltryptamine. Anal. Chem. 2025, 97 (11), 6163–6174.

(10) Barker, S. A.; Borjigin, J.; Lomnicka, I.; Strassman, R. LC/MS/MS Analysis of the Endogenous Dimethyltryptamine Hallucinogens, Their Precursors, and Major Metabolites in Rat Pineal Gland Microdialysate. Biomed. Chromatogr. 2013, 27 (12), 1690–1700.

(11) Hashima, Y.; Takahashi, T.; Ishikawa, Y.; Uraoka, Y. Development of High-Reliability and -Stability Chemical Sensors Based on an Extended-Gate Type Amorphous Oxide Semiconductor Thin-Film Transistor. ACS Appl. Electron. Mater. 2020, 2 (2), 405–408.

(12) Nishitani, S.; Sakata, T. Enhancement of Signal-to-Noise Ratio for Serotonin Detection with Well-Designed Nanofilter-Coated Potentiometric Electrochemical Biosensor. ACS Appl. Mater. Interfaces 2020, 12 (13), 14761–14769.

(13) Zhou, Q.; Sasaki, Y.; Ohshiro, K.; Fan, H.; Montagna, V.; Gonzato, C.; Haupt, K.; Minami, T. An Organic Transistor for the Selective Detection of Tropane Alkaloids Utilizing a Molecularly Imprinted Polymer. J. Mater. Chem. B 2022, 10 (35), 6808–6815.

(14) Kajisa, T.; Li, W.; Michinobu, T.; Sakata, T. Well-Designed Dopamine-Imprinted Polymer Interface for Selective and Quantitative Dopamine Detection among Catecholamines Using a Potentiometric Biosensor. Biosens. Bioelectron. 2018, 117, 810–817.

(15) Tarasov, A.; Gray, D. W.; Tsai, M.-Y.; Shields, N.; Montrose, A.; Creedon, N.; Lovera, P.; O'Riordan, A.; Mooney, M. H.; Vogel, E. M. A Potentiometric Biosensor for Rapid On-Site Disease Diagnostics. Biosens. Bioelectron. 2016, 79, 669–678.
